# Discovery and Biochemical Characterization of a Methanol Dehydrogenase From *Lysinibacillus xylanilyticus*

**DOI:** 10.3389/fbioe.2020.00067

**Published:** 2020-02-14

**Authors:** Jin-Young Lee, Sung-Hyun Park, So-Hyung Oh, Jin-Ju Lee, Kil Koang Kwon, Su-Jin Kim, Minjeong Choi, Eugene Rha, Hyewon Lee, Dae-Hee Lee, Bong Hyun Sung, Soo-Jin Yeom, Seung-Goo Lee

**Affiliations:** ^1^Synthetic Biology and Bioengineering Research Center, Korea Research Institute of Bioscience and Biotechnology, Daejeon, South Korea; ^2^Department of Biosystems and Bioengineering, KRIBB School of Biotechnology, University of Science and Technology, Daejeon, South Korea; ^3^School of Biological Sciences and Technology, Chonnam National University, Gwangju, South Korea

**Keywords:** methanol dehydrogenase, rational enzyme engineering, *Lysinibacillus xylanilyticus*, methylotrophy, methanol oxidation

## Abstract

Bioconversion of C1 chemicals such as methane and methanol into higher carbon-chain chemicals has been widely studied. Methanol oxidation catalyzed by methanol dehydrogenase (Mdh) is one of the key steps in methanol utilization in bacterial methylotrophy. In bacteria, few NAD^+^-dependent Mdhs have been reported that convert methanol to formaldehyde. In this study, an uncharacterized Mdh gene from *Lysinibacillus xylanilyticus* (Lxmdh) was cloned and expressed in *Escherichia coli*. The maximum alcohol oxidation activity of the recombinant enzyme was observed at pH 9.5 and 55°C in the presence of 10 mM Mg^2+^. To improve oxidation activity, rational approach-based, site-directed mutagenesis of 16 residues in the putative active site and NAD^+^-binding region was performed. The mutations S101V, T141S, and A164F improved the enzyme’s specific activity toward methanol compared to that of the wild-type enzyme. These mutants show a slightly higher turnover rate than that of wild-type, although their *K*_M_ values were increased compared to that of wild-type. Consequently, according the kinetic results, S101, T141, and A164 positions may related to the catalytic activity in the active site for methanol dehydrogenation. It should be further studied other mutant variants with high activity for methanol. In conclusion, we characterized a new Lxmdh and its variants that may be potentially useful for the development of synthetic methylotrophy in the future.

## Introduction

Methanol dehydrogenase (Mdh) catalyzes the interconversion of methanol and formaldehyde via an oxidation-reduction reaction. In methylotrophs, methanol is oxidized by Mdh; efficient methanol oxidation and concomitant formaldehyde assimilation and dissimilation is critical for the growth and energy generation of these organisms. In this regard, Mdh is a crucial enzyme for bioconversion of valuable multi-carbon chemicals from C1 chemicals through methylotrophy or non-methylotrophy such as *Escherichia coli*.

In methylotrophic bacteria, Mdhs are classified into three groups based on their electron acceptors as follows: pyrroloquinoline quinone (PQQ)-dependent Mdh, O_2_-dependent alcohol oxidase, and NAD^+^-dependent Mdh. PQQ-dependent Mdh is a periplasmic enzyme containing redox cofactor PQQ, which is synthesized through a complex biosynthetic pathway involving oxidation. Alcohol oxidase in the peroxisome of yeast can oxidize alcohols including methanol to aldehyde and hydrogen peroxide, which are highly toxic to cells. These two types of Mdhs require oxygen and move to specific cellular locations for their proper function. However, NAD^+^-dependent Mdh is located in the cytoplasmic region and functions under both aerobic and anaerobic conditions. Therefore, NAD^+^-dependent Mdh may be the best candidate for synthetic methylotrophy because it can perform its function under both aerobic and anaerobic conditions and generate reducing equivalents (NADH), which can help promote strain growth (Zhang et al., 2017).

Methanol utilization studies of recombinant *E. coli* as synthetic methylotrophs containing NAD^+^-dependent Mdhs from *Bacillus stearothermophilus*, *Bacillus methanolicus*, and *Cupriavidus necator* have been previously reported (Muller et al., 2015; Wu et al., 2016; Whitaker et al., 2017). However, recombinant *E. coli* as synthetic methylotroph strains cannot utilize methanol as the sole carbon source because of their low affinity for methanol or delta G energy (Nguyen et al., 2016). Although Mdhs and the ribulose monophosphate pathway (Rump) can be successfully assembled in non-native methylotrophs, methanol consumption as the sole carbon source in these cells have not yet been reported (Whitaker et al., 2017; Chen et al., 2018). The catalytic activity of Mdhs of *B. methanolicus* for methanol oxidation was dramatically enhanced by an endogenous activator protein (ACT); however, the detailed mechanism for Mdh activation remains unclear (Hektor et al., 2002; Krog et al., 2013). To enable metabolic engineering to assimilate methanol as a carbon source, a new Mdh without ACT and with high activity under mesophilic or thermophilic conditions is needed. Recently, only one ACT-independent Mdh and its mutants from *C. necator* N-1 as gram-negative bacteria have been reported (Wu et al., 2016) and introduced into *E. coli* for methanol assimilation (Chen et al., 2018). However, Mdh from *C. necator* N-1 require a small amount of yeast extract as a carbon source for methanol assimilation. Thus, ACT-independent Mdh enzymes with high activity for methanol should be further studied.

In this study, Mdh from *Lysinibacillus xylanilyticus* as a gram-positive bacterium was characterized as an ACT-independent Mdh, which has not been previously reported. Based on the homology modeling structure of Lxmdh, we developed a rational protein engineering strategy to increase the methanol oxidation activity and successfully obtained Lxmdh variants with enhanced activity. We developed a new Mdh that can react with C1 chemicals in synthetic microorganisms.

## Materials and Methods

### Chemicals and Materials

All chemical reagents used in this study were purchased from Sigma-Aldrich (St. Louis, MO, United States). Oligonucleotides and gene synthesis reagents were provided by Macrogen (Seoul, South Korea). Restriction endonucleases, polymerases, and DNA cloning kits were purchased from New England Biolabs (Ipswich, MA, United States). DNA preparation and manipulation techniques were carried out according to standard protocols for molecular biology. The kits for PCR product purification, gel extraction, and plasmid preparation were purchased from Promega (Madison, WI, United States). Profinia^TM^ purification kits and all materials for SDS-PAGE were purchased from Bio-Rad (Hercules, CA, United States).

### Gene Cloning and Site-Directed Mutagenesis of Lxmdh

The gene (1206 base pairs) encoding *lxmdh* was obtained using genomic DNA isolated from *Lysinibacillus xylanilyticus* KCTC 13423. *E. coli* C2566 (New England Biolabs) and the pET-28a (+) plasmid (Novagen, Merck KGaA, Darmstadt, Germany) were used as host cells and the expression vector, respectively. The Lxmdh coding region was cloned between the T7 promoter and terminator in the pET-28a (+) plasmid containing an N-terminal His_6_ tag. Forward (5′-atcgcatatgtcagacgttctaaagcaatttg-3′) and reverse (5′-atcgctcgagttaagaaagtgcgacag-3′) primers were designed to introduce the *Nde*I and *Xho*I restriction sites (underlined), respectively. The PCR product was subcloned into the pET-28a(+) plasmid digested with the same restriction enzymes and then transformed into *E. coli* C2566. Site-directed mutagenesis was performed using the Quick-Change kit and protocol (Stratagene, San Diego, CA, United States). The constructed plasmid was confirmed to have the correct sequence by Sanger sequencing (Macrogen).

### Lxmdh Purification

Lxmdh expressing cells were harvested from the culture broth and disrupted on ice by ultrasonication (Thermo Fisher Scientific, Waltham, MA, United States) in buffer A (50 mM sodium monophosphate, 300 mM NaCl, 10 mM imidazole, and 0.1 mM phenylmethylsulfonyl fluoride as a protease inhibitor). Unbroken cells and cell debris were removed by centrifugation at 14,000 rpm for 10 min at 4°C, and the supernatants were filtered through a 0.45-μm filter and applied to an immobilized metal affinity chromatography (IMAC) column (Bio-Rad) equilibrated with buffer A. Supernatants collected from the lysates were loaded into the Profinia^TM^ Purification System (Bio-Rad). Supernatants were loaded onto a 1-mL IMAC cartridge and washed twice with 5 and 10 mM imidazole buffer A. Proteins were eluted with 250 mM imidazole in buffer A. Imidazole and other salts were removed and changed with 50 mM CHES buffer (pH 9.5) using a desalting cartridge. The resulting solution was used as the purified Lxmdh enzyme. The protein concentration was quantified by the standard Bradford method (Bradford, 1976). The purified proteins were confirmed by SDS-PAGE.

### Molecular Mass Determination of Lxmdh

The subunit molecular mass of Lxmdh was evaluated by SDS-PAGE under denaturing conditions using a pre-stained ladder (Bio-Rad) as reference proteins. All protein bands were stained with Coomassie blue for visualization. The native molecular mass of the enzyme was determined by gel-filtration chromatography on a Superose 12 10/300 GL column (GE Healthcare, Little Chalfont, United Kingdom). The purified enzyme was applied to the column and eluted with 25 mM Tris–HCl (pH 7.4) buffer containing 200 mM NaCl at a flow rate of 1 mL/min. The column was calibrated with thyroglobulin (669 kDa), apoferritin (443 kDa), β-amylase (200 kDa), alcohol dehydrogenase (150 kDa), and albumin (66 kDa) as reference proteins (Sigma-Aldrich), and the native molecular mass of the enzyme was calculated based on reference protein migration.

### Effects of pH, Temperature, and Metal Ions

To examine the effect of pH on Lxmdh activity, the pH was varied between 50 mM HEPES buffer [3-(*N*-morpholino)propanesulfonic acid; pH 7.5–8.0], 50 mM EPPS buffer [4-(2-hydroxyethyl)-1-piperazinepropanesulfonic acid; pH 8.0–8.5] and CHES buffer [2-(cyclohexylamino)ethanesulfonic acid: pH 8.5–10.0] containing 5 mM Mg^2+^ and 3 mM NAD. To investigate the effect of temperature on Lxmdh enzyme activity, the temperature was varied from 35 to 70°C. To evaluate the effect of metal ions on enzyme activity, an enzyme assay was conducted after treatment with 10 mM EDTA at 4°C for 1 h or after adding 1 mM concentration of each metal ion (Mn^2+^, Zn^2+^, Cu^2+^, Ni^2+^, Co^2+^, Mg^2+^, Ca^2+^, or Fe^2+^). The reactions were performed in 50 mM CHES buffer (pH 9.5) containing each metal ion at 55°C.

### Measurement of Activity and Determination of Kinetic Parameters of Wild-Type and Mutant Enzymes

Mdh activity assays were carried out in a 100-μL assay mixture containing 50 mM CHES buffer (pH 9.5), 5 mM of Mg^2+^, 3 mM NAD^+^, and 500 mM of methanol, ethanol, propanol, and butanol at 55°C. One unit (U) of Lxmdh was defined as the amount of enzyme required to produce 1 nmol of NADH per min at 55°C and pH 9.5. In kinetic analysis, various amounts of substrates (0.5–800 mM) were incubated in 50 mM CHES buffer (pH 9.5) containing 5 mM Mg^2+^ and Lxmdh enzyme at 55°C for 5 min. The kinetic parameters were determined by fitting the data to the Michaelis–Menten equation.

### Comparative Homology Modeling

Homology modeling of Lxmdh was carried out using Discovery Studio 3.1 (Accelrys, San Diego, CA, United States) based on the X-ray structure of lactaldehyde dehydrogenase (PDB; 5BR4) derived from *E. coli*. Homology searches and sequence alignment were conducted using sequence analysis and multiple sequence alignment modules, respectively. Five models were generated based on the alignment of the target sequence with its template using the MODELLER software program (Marti-Renom et al., 2000) by applying the default model-building routine model with fast refinement. This procedure allows for selection of the best model from among several candidates and variability among models can be used to evaluate model reliability. Energy minimization was applied using a consistent valence force field and DS CHARMm with the steepest descent and conjugated gradient algorithms. Model quality was analyzed with PROCHECK software (Laskowski et al., 1993). Methanol was docked as the ligand in the Lxmdh model using AutoDock Vina (Trott and Olson, 2010). Docking pocket residues were searched using the Pck pocket detection program^1^. The lowest energy conformation was selected for further analyses.

## Results and Discussion

### Gene Cloning, Purification, and Molecular Mass of Lxmdh

**FIGURE 1:**
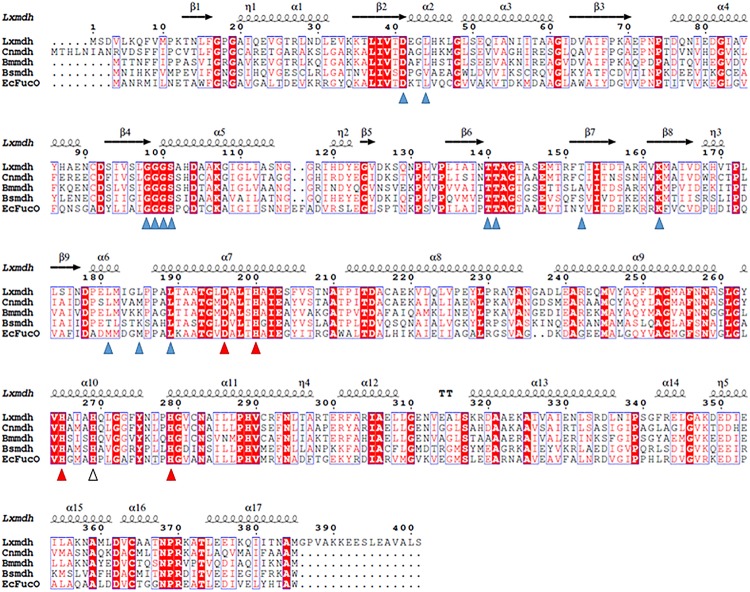
Sequence alignment of Lxmdh and other type III alcohol dehydrogenases. Amino acid sequences of Lxmdh, *L. xylanilyticus* Mdh; Cnmdh, *C. necator* N-2 Mdh; Bmmdh, *B. methanolicus* Mdh; EcFucO, *E. coli* lactaldehyde dehydrogenase. The NAD-binding site (blue triangle) and metal-binding site (red triangle) are indicated.

The gene (1206 base pairs) encoding alcohol dehydrogenase from *L. xylanilyticus* was cloned and expressed in *E. coli*. The expressed enzyme contained a 6-histidine tag at the N-terminus consisting of 401 amino acid residues and showed alcohol dehydrogenase activity by converting alcohol to the corresponded aldehyde. Sequence alignment of Lxmdh revealed that 59% (222/378), 42% (160/381), 62% (240/385), and 40% (154/381) amino acid sequence identity with Mdh from *B. methanolicus*, *B. stearothermophilus*, *C. necator* and to lactaldehyde reductase (FucO) from *E. coli*, respectively (Figure 1).

**FIGURE 2:**
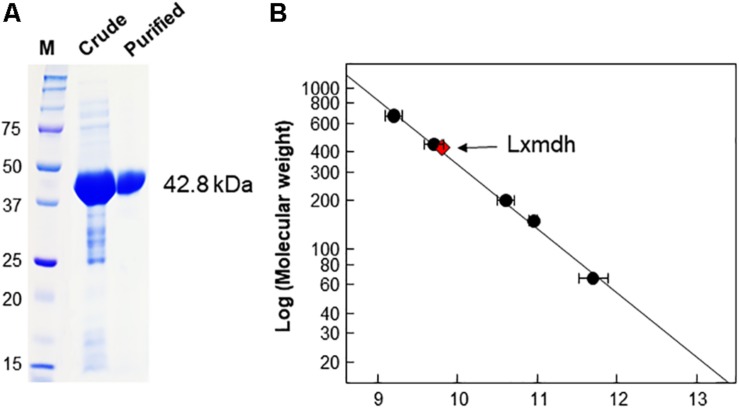
SDS-PAGE analysis and determination of the molecular mass of Lxmdh. **(A)** SDS-PAGE of Lxmdh. Pre-stained marker protein (250, 150, 100, 75, 50, 37, and 25 kDa), crude extract, and purified enzyme were loaded. **(B)** Determination of the molecular mass of Lxmdh by gel-filtration chromatography. Reference proteins indicated by closed circles are thyroglobulin (669 kDa), apoferritin (443 kDa), β-amylase (200 kDa), alcohol dehydrogenase (150 kDa), and albumin (66 kDa). Lxmdh is represented by a red diamond.

Lxmdh showed high sequence similarity with other alcohol dehydrogenase and was classified into the iron-containing alcohol dehydrogenase enzyme family (type III Adhs), as confirmed by BLAST search (NCBI). The structures of some enzymes in this family have been solved, revealing dimer or decamer forms (Montella et al., 2005; Marcal et al., 2009; Moon et al., 2011). To confirm the size of quaternary structure of Lxmdh, soluble protein was purified from the crude extract obtained from harvested cells by IMAC purification system. The subunit molecular mass of the purified Lxmdh according to SDS-PAGE was approximately 42.8 kDa (Figure 2A). The molecular mass of the native enzyme was approximately 420 kDa and formed a decamer as determined by gel filtration chromatography using a Sephacryl S-300 HR 16/60 column and based on the reference protein masses (Figure 2B).

The type III Adh enzymes have some common structural features as follows: the N- and C-terminal domains of these protein are separated by a deep cleft, and the cofactor, NAD(H), is in the cleft with a glycine-rich motif (Figure 1). In the active site, a specific metal ion such as Fe^2+^, Zn^2+^, or Mg^2+^ is tetrahedrally coordinated through 4 amino acid residues (3 His and Asp) and mediates the metal-dependent dehydrogenation catalytic reaction (Figure 1). Accordingly, all of these residues related to cofactor binding and metal binding are well conserved among the type III Adh enzymes (Obradors et al., 1998; Montella et al., 2005; Marcal et al., 2009; Moon et al., 2011; Ochsner et al., 2014). The oligomeric state of the enzyme is related to enzyme stability and the allosteric regulation of its activity (Marcal et al., 2009).

### Effects of Metal Ions, pH, and Temperature

**FIGURE 3:**
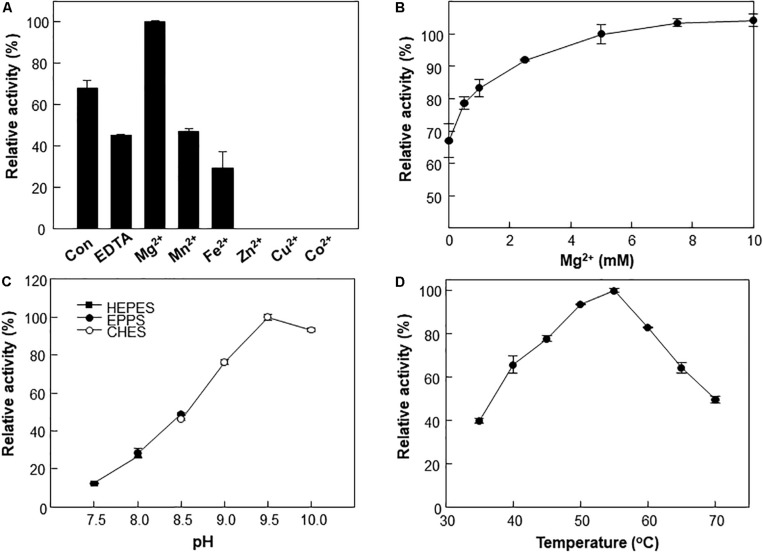
Effects of pH, temperature and metal ions on Lxmdh activity. **(A)** Effects of different metal ions on Lxmdh activity. Reactions were performed in 50 mM CHES buffer (pH 9.5) containing 3 mM NAD^+^ and 1 mM of each metal ion at 55°C for 5 min. **(B)** Effects of Mg^2+^ concentration on Lxmdh activity. **(C)** pH: reactions were performed in 50 mM HEPES buffer (closed square) or 50 mM EPPS (closed circle), 50 mM CHES buffer (opened circle) containing 3 mM NAD^+^ and 5 mM Mg^2+^ at 55°C for 5 min. **(D)** Temperature: reactions were performed in 50 mM CHES buffer (pH 9.5) containing 3 mM NAD^+^ and 5 mM Mg^2+^ with the enzyme for 5 min.

A metal ion is crucial for the reaction of type III Adhs. Enzyme activity is generally increased by supplementation with Fe^2+^ or Mn^2+^ ions and inhibited by supplementation with Zn^2+^, Cu^2+^, or Co^2+^ ions (Sridhara et al., 1969; Montella et al., 2005). The metal ions are involved in cofactor binding and may influence enzymatic activity (Hektor et al., 2002). We examined the effects of various divalent metals on the methanol oxidation activity of Lxmdh. Among the metal ions tested, 10 mM Mg^2+^ had the strongest positive effects on the methanol oxidation activity of Lxmdh (Figures 3A,B). This activity was slightly decreased by the Mn^2+^ or Fe^2+^ ions and lost with the Zn^2+^, Cu^2+^, and Co^2+^ ions. Enzyme stability was slightly affected by high concentrations of Mg^2+^, which resulted in aggregation. Thus, all subsequent experiments were performed in the presence of 5 mM Mg^2+^ as a cofactor.

The effects of pH and temperature on methanol oxidation activity of Lxmdh were also investigated, and maximum activity was observed at pH 9.5 and 55°C (Figures 3C,D). These results are similar to those previously reported for other Mdh from *B. methanolicus* and *C. necator* (Krog et al., 2013; Muller et al., 2015; Wu et al., 2016).

**FIGURE 4:**
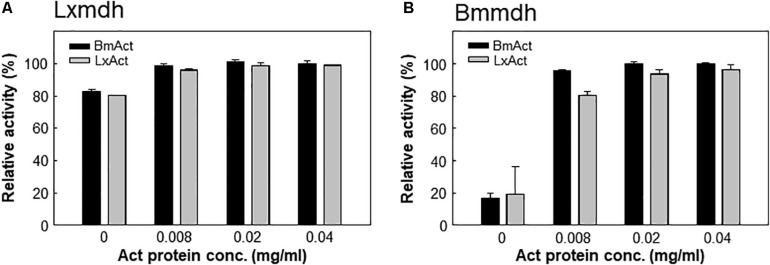
Lxmdh insensitivity to activation effect. Lxmdh **(A)** and Bmmdh **(B**, control) activity was measured in the presence of two activator proteins (BmAct, LxAct). The specific activity was measured in presence of activator protein under conditions of pH 9.5 and 55°C.

Most type III Mdhs are activated by Act protein, which results in improvement in the *V*_max_ and *K*_M_ for methanol (Ochsner et al., 2014; Muller et al., 2015). However, Lxmdh was insensitive to the activation effect by Act protein (Figures 4A,B), similar to Cnmdh which was previously characterized as an activator-independent Mdh (Wu et al., 2016). Although the activator protein may alter the ping-pong type of reaction mechanism of Mdhs to the ternary-complex mechanism to increase the reaction rate, the detailed mechanism of Mdh activation is unclear (Arfman et al., 1997; Hektor et al., 2002).

### Substrate Specificity of Lxmdh

Substrate specificity is thought to be determined by the structural features and interaction between substrates and both the nicotinamide ring of cofactor and surrounding residues in the catalytic pocket. The catalytic pocket surrounded several hydrophobic residues and the nicotinamide ring is inside of the pocket. Accordingly, access of the substrate to the catalytic pocket is regulated by the orientation of nicotinamide ring and surrounding residues via charged/hydrophobic interactions.

To understand the substrate specificity of Lxmdh, specific activity was investigated for various alcohols such as methanol (C1), ethanol (C2), n-propanol (C3), and butanol (C4). Interestingly, Lxmdh exhibited relatively high activity toward methanol among the alcohols tested (Table 1). In a previous study, all Mdhs show low methanol oxidation activity compared to the activity toward C2–C4 alcohols. In the case of Mdh from *C. necator* (Cnmdh), the enzyme activity for methanol is considerably low compared to that toward ethanol as a substrate (Wu et al., 2016). However, Lxmdh showed comparable dehydrogenase activity toward methanol as other alcohol substrates, indicating that it has relatively high substrate specificity toward methanol compared to the Mdhs from Bmmdh, Bsmdh, and Cnmdh. The enzyme activities for C1–C4 alcohol were tested with previously reported Mdhs to compare their activity (Supplementary Figure S1). According to the results, among the reported Mdhs, Lxmdh showed comparable dehydrogenase activity toward methanol.

**TABLE 1:** Kinetic parameters for Lxmdh with C1–C4 alcohols as substrates.

Substrate	*V*_max_ (mU/mg)	*k*_cat_ (s^–^^1^)	*K*_M_ (mM)	*k*_cat_/*K*_M_ (M^–^^1^ s^–^^1^)
Methanol	302.7 ± 16.9	0.21 ± 0.01	3.23 ± 1.05	66.8
Ethanol	652.4 ± 11.9	0.46 ± 0.00	0.25 ± 0.03	1861.8
n-Propanol	777.8 ± 32.9	0.55 ± 0.02	1.09 ± 0.25	509.1
Butanol	734.2 ± 37.3	0.52 ± 0.02	0.50 ± 0.14	1047.6
NAD^+^	531.2 ± 10.7	0.37 ± 0.00	0.23 ± 0.01	1647.8

*Mdh assay to determine *K*_*M*_ for different alcohols were performed using various alcohol concentrations and 5 mM NAD^+^ at 55°C and pH 9.5. To determine the kinetic parameters of NAD^+^, 500 mM ethanol was used.*

The Michaelis–Menten constant (*K*_M_), turnover rate (*k*_cat_), and catalytic efficiency (*k*_cat_/*K*_M_) of the enzyme toward C1–C4 alcohols were also determined (Table 1). The *K*_M_ and *k*_cat_ value for methanol were 3.23 mM and 0.22 s^–1^, respectively. The *K*_M_ value is quite low, as compared to that of Cnmdh (Wu et al., 2016) and those of Mdh and Mdh2 derived from *B. methanolicus* (Ochsner et al., 2014), which are in the range of 9–132 mM. Therefore, it was assumed that the Lxmdh would be a good candidate for oxidation of methanol.

### Rational Design Strategy for Enhancing the Methanol Oxidation Activity of Lxmdh

**FIGURE 5:**
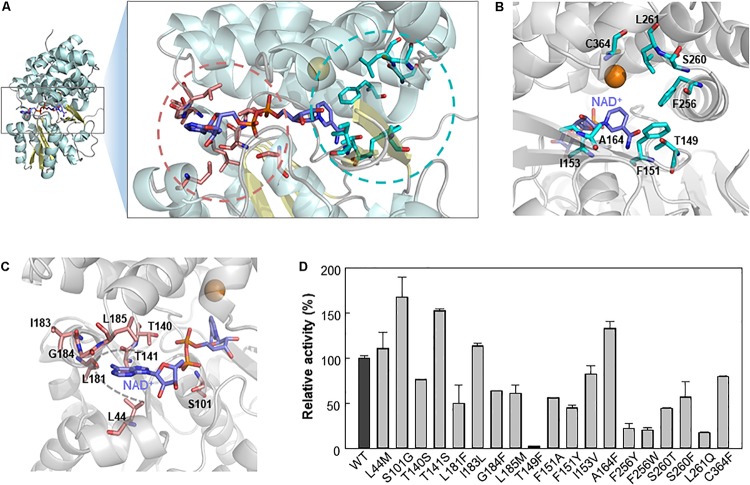
Selection of mutable residues to improve Lxmdh activity toward methanol. **(A)** Selected residues for mutation study. **(B)** After molecular docking simulation with the homology model structure of Lxmdh, the selected residues that can interact with methanol ligand in the catalytic pocket region were selected and represented as a stick (pale blue). **(C)** Mutable residues that interact with the cofactor NAD^+^ (purple), in the adenine-binding pocket, and pyrophosphate interaction residues were selected and as represented as a stick (pale red). **(D)** Comparison of relative activity toward methanol in Lxmdh mutant variants. Each mutant variant of Lxmdh was constructed by site-directed mutagenesis. The methanol dehydrogenase activity of each of mutant variant was analyzed at pH 9.5 and 55°C.

We further analyzed Lxmdh by introducing site-directed mutations in the catalytic pocket and NAD(H) binding region through structure-based analysis to improve enzymatic activity. Although structural changes in the protein have occurred during evolution (Lesk and Chothia, 1980), the active sites of related proteins have very similar geometries (Lesk and Chothia, 1982; Read et al., 1984). The active site structure in a protein may be a good model for those in related proteins even if the overall sequence homologies are low (Chothia and Lesk, 1986). The crystal structure (PBD number, 5BR4) of *E. coli* lactaldehyde reductase (FucO) was chosen as a template of homology model (40% sequence identity with Lxmdh). The active site in the homology model of Lxmdh was determined based on multiple-sequence alignment with the crystal structure of FucO (Supplementary Figure S2). To identify the specific residues involved in substrate binding activity, we performed a ligand-docking study of methanol with the homology model (Supplementary Figure S3). From the docking results, we selected eight residues (T149, F151, I153, A164, F256, S260, L261, and C364) within 4.5 Å of the center of the docked substrate as candidates for studying enzyme activity (Figures 5A,B and Supplementary Figure S3).

It has been reported that NAD(P)H-dependent enzymes show improved enzymatic activity following engineering of the NAD(P)H binding affinity, particularly in the adenine binding pocket (Cahn et al., 2016), and NAD(P)^+^ binding strength may alter the catalytic efficiency of the enzyme. To identify the mutable residues in the NAD^+^-binding pocket in Lxmdh, we searched for residues within 6 Å of the center of the adenine ring of cofactor and selected 8 residues (L44, S101, T140, T141, L181, I183, G184, and L185) as candidates (Figures 5A,C). Sixteen residues were investigated to identify those with positive influence on enzymatic activity toward methanol. First, we conducted sequence alignment of lxmdh comparing with other type III alcohol dehydrogenase, identified the frequencies of amino acids at the each of residues. The selected residues located in the NAD^+^ binding pocket were replaced with evolutionary relevant amino acids or those with similar character, and the selected residues deduced from docking simulation were replaced with the specific amino acids. The specific activities of the mutants were determined to identify which residues increase the specific activity for methanol oxidation. As a result, among the mutant variants, the S101G, T141S, and A164F mutants showed enhanced catalytic activity toward methanol (Figure 5D). In contrast, the T149F mutant lost nearly all-catalytic activity toward alcohols including methanol, indicating that the bulky residue at position 149 had a negative effect on methanol oxidation. Residue A164 located in the substrate binding pocket may be related to substrate affinity or catalytic activity (Figure 5B), and residues S101 and T141 located next to the pyrophosphate group of NAD^+^ may affect NAD^+^ binding status (Figure 5C).

**FIGURE 6:**
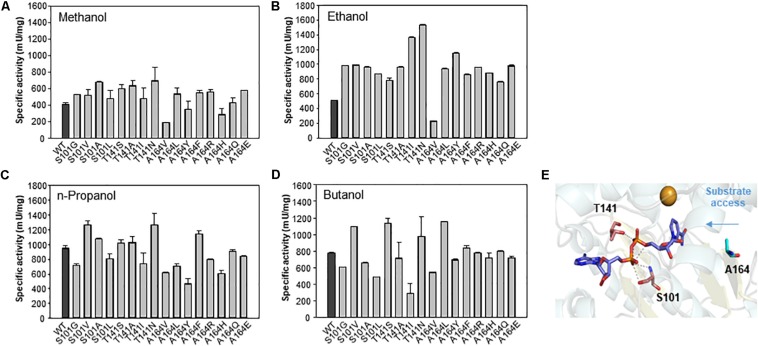
Comparison of specific activities of wild-type Lxmdh with S101, T141, and A164 mutant variants. The specific activities toward C1–C4 alcohols **(A–D)** were measured in 50 mM CHES buffer (pH 9.5) containing 3 mM NAD^+^ and 5 mM Mg^2+^ with the enzyme at 55 °C for 5 min. **(E)** S101, T141, and A164 are represented as a stick in the homology model.

To determine the role of the S101, T141, and A164 positions, we further substituted the residues with other amino acids. A total of 16 variants were obtained, and specific activity was measured with C1–C4 alcohols as substrates (Figure 6). As a result, the S101G, T141S, and A164F mutants showed increased activity toward C1 and C2 alcohols. Mutants S101V, T141S, and A164F showed a higher turnover rate compared to that of wild type, although their *K*_M_ values were increased compared to that of wild-type. These results indicated that the S101, T141, and A164 residues might be involved in the catalysis for methanol dehydrogenation (Table 2). We also determined the NAD^+^ binding affinity in the three mutants (S101V, T141S, and A164F), and these variants showed higher *K*_M_ values for NAD^+^ compared to wild-type Lxmdh (Supplementary Figure S4). These results suggest that the lower affinity for NAD^+^ in S101V, T141S, and A164F may influences catalytic activity. In this study, we characterized a new Mdh and its variants for methanol oxidation. To obtain variants with further improved *K*_M_, additional mutational studies such as random mutagenesis should be conducted with low concentration of substrates during the screening procedure. To apply Mdhs to synthetic methylotrophy, detailed studies of engineered enzymes, such as thermal stability and activity under physiological conditions, must be performed. Mdhs with improved affinity toward small amounts of methanol should be developed in future studies for efficient oxidation of methanol.

**TABLE 2:** Kinetic parameters for Lxmdh mutants with C1–C3 alcohols as substrates.

Lxmdh variants	Methanol				Ethanol				n-Propanol			
	*V*_max_ (mU/mg)	*k*_cat_ (s^–1^)	*K*_M_ (mM)	*k*_cat_/*K*_M_ (M^–1^ s^–1^)	*V*_max_ (mU/mg)	*k*_cat_ (s^–1^)	*K*_M_ (mM)	*k*_cat_/*K*_M_ (M^–1^ s^–1^)	*V*_max_ (mU/mg)	*k*_cat_ (s^–1^)	*K*_M_ (mM)	*k*_cat_/*K*_M_ (M^–1^ s^–1^)
WT	302.7 ± 16.89	0.21 ± 0.01	3.23 ± 1.05	66.8	652.4 ± 11.98	0.46 ± 0.00	0.25 ± 0.03	1861.8	777.8 ± 32.97	0.55 ± 0.02	1.09 ± 0.25	509.1
S101V	342.3 ± 21.67	0.24 ± 0.01	10.35 ± 3.87	23.6	827.2 ± 28.70	0.59 ± 0.02	17.38 ± 2.73	33.9	444.9 ± 17.88	0.31 ± 0.01	9.45 ± 1.49	33.5
T141S	462.9 ± 57.60	0.33 ± 0.04	51.24 ± 23.95	6.4	916.3 ± 24.76	0.65 ± 0.01	1.38 ± 0.23	473.7	972.6 ± 30.32	0.69 ± 0.02	1.82 ± 0.34	381.2
A164F	475.3 ± 50.72	0.33 ± 0.03	36.83 ± 15.82	9.2	872.5 ± 30.73	0.62 ± 0.02	1.19 ± 0.25	523.1	1111 ± 29.69	0.79 ± 0.02	2.33 ± 0.36	340.2

## Conclusion

Development of synthetic methylotrophy is becoming an important research area because of valuable chemicals can be produced through C1 assimilation. Mdh is the first enzyme involved in methanol oxidation, and thus is important for successive methanol-dependent growth in methylotrophy. Here, Mdh from *L. xylanyliticus* was characterized and engineered. The Lxmdh has higher activity and lower *K*_M_ toward methanol compared to that of other type III Mdhs, showed independent activity by Act protein. To determine active site, we conducted mutation analysis at specific residues in the active pocket and NAD^+^-binding site, deduced by a rational approach using a homology model structure of Lxmdh. This finding may be potentially useful for the development of synthetic methylotrophy in the future.

## Data Availability Statement

The raw data supporting the conclusions of this article will be made available by the authors, without undue reservation, to any qualified researcher.

## Author Contributions

J-YL, BS, S-JY, and S-GL: conceptualization. J-YL, S-HP, and J-JL: methodology. J-YL, S-HO, and S-JY: validation. KK, S-JK, and MC: formal analysis. J-YL, S-HP, S-HO, J-JL, and ER: data curation. J-YL and S-JY: writing – original draft preparation and visualization. ER, HL, D-HL, BS, and S-GL: writing – review and editing. S-JY and S-GL: supervision, project administration, and funding acquisition.

## Conflict of Interest

The authors declare that the research was conducted in the absence of any commercial or financial relationships that could be construed as a potential conflict of interest.

## Footnotes

1 http://schwarz.benjamin.free.fr/Work/Pck/home.htm

## Abbreviations

ACT: endogenous activator protein
IMAC: immobilized metal affinity chromatography
Lxmdh: *Lysinibacillus xylanilyticus* derived methanol dehydrogenase
Mdh: methanol dehydrogenase
PQQ: pyrroloquinoline quinone.
