# Supplementary material for: Discovery and Biochemical Characterization of a Methanol Dehydrogenase From Lysinibacillus xylanilyticus
Source: Front Bioeng Biotechnol. 2020 Feb 14;8:67. doi: 10.3389/fbioe.2020.00067 (PMC7033420; doi:10.3389/fbioe.2020.00067)
Supplement: Supplementary file 1 [file Presentation_1.PPTX]

## Slide 1
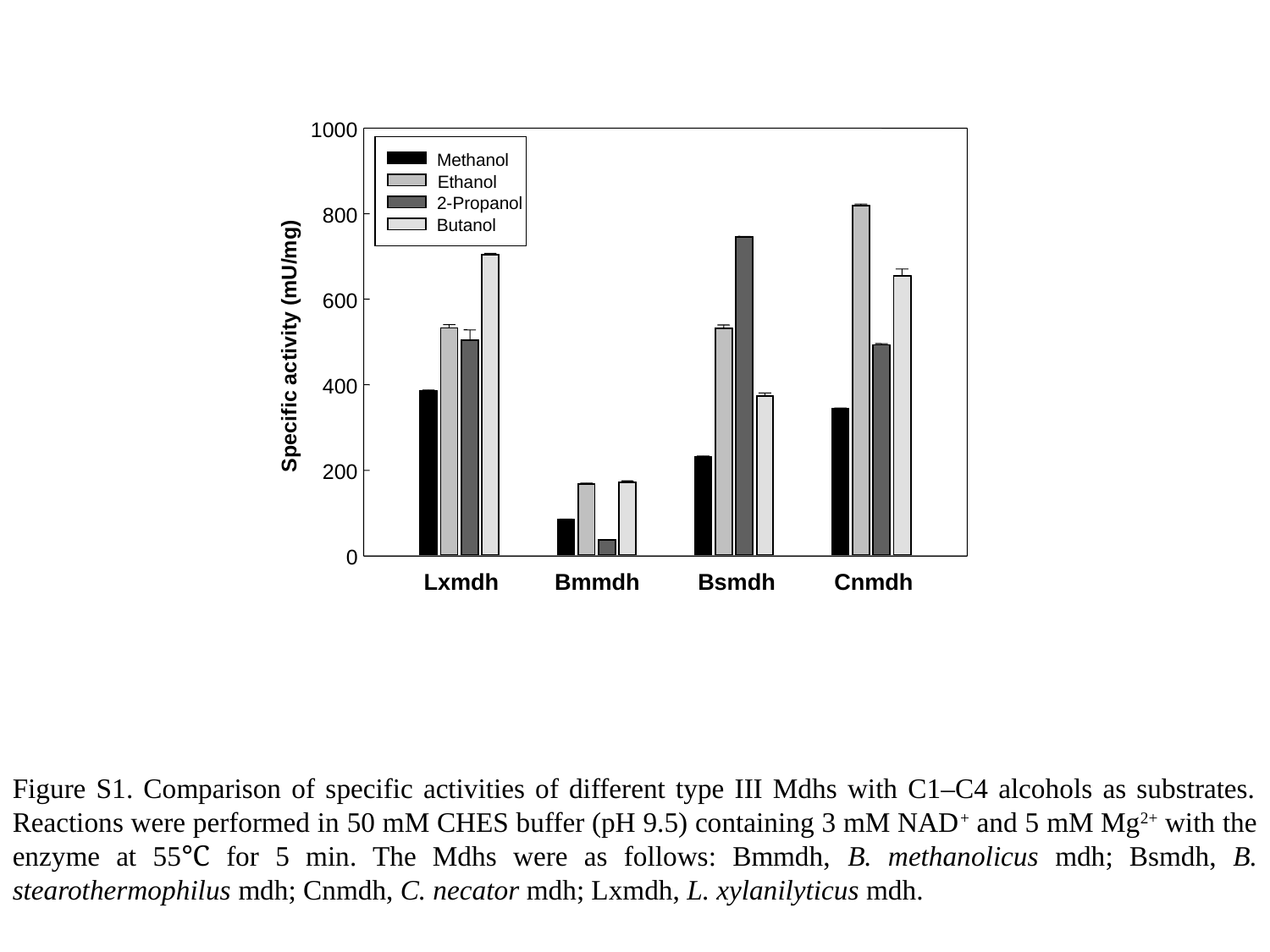

1000
800
600
400
200
0
Lxmdh
Bmmdh
Bsmdh
Cnmdh
Methanol
Ethanol
2-Propanol
Butanol
Specific activity (mU/mg)
Figure S1. Comparison of specific activities of different type III Mdhs with C1–C4 alcohols as substrates. Reactions were performed in 50 mM CHES buffer (pH 9.5) containing 3 mM NAD+ and 5 mM Mg2+ with the enzyme at 55℃ for 5 min. The Mdhs were as follows: Bmmdh, B. methanolicus mdh; Bsmdh, B. stearothermophilus mdh; Cnmdh, C. necator mdh; Lxmdh, L. xylanilyticus mdh.

## Slide 2
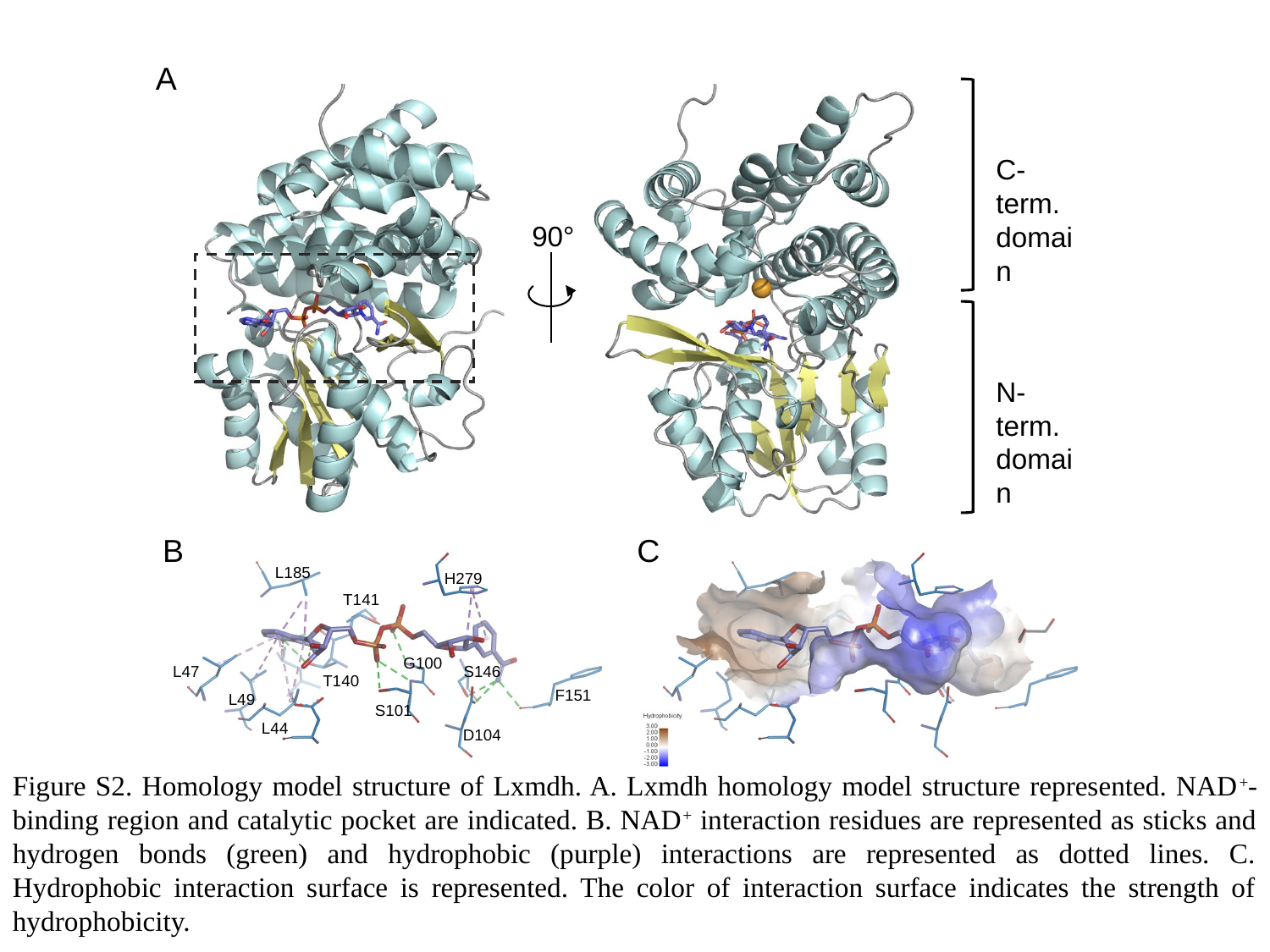

A
C-term. domain
90°
N-term. domain
B
C
L185
H279
T141
G100
L47
S146
T140
F151
L49
S101
L44
D104
Figure S2. Homology model structure of Lxmdh. A. Lxmdh homology model structure represented. NAD+-binding region and catalytic pocket are indicated. B. NAD+ interaction residues are represented as sticks and hydrogen bonds (green) and hydrophobic (purple) interactions are represented as dotted lines. C. Hydrophobic interaction surface is represented. The color of interaction surface indicates the strength of hydrophobicity.

## Slide 3
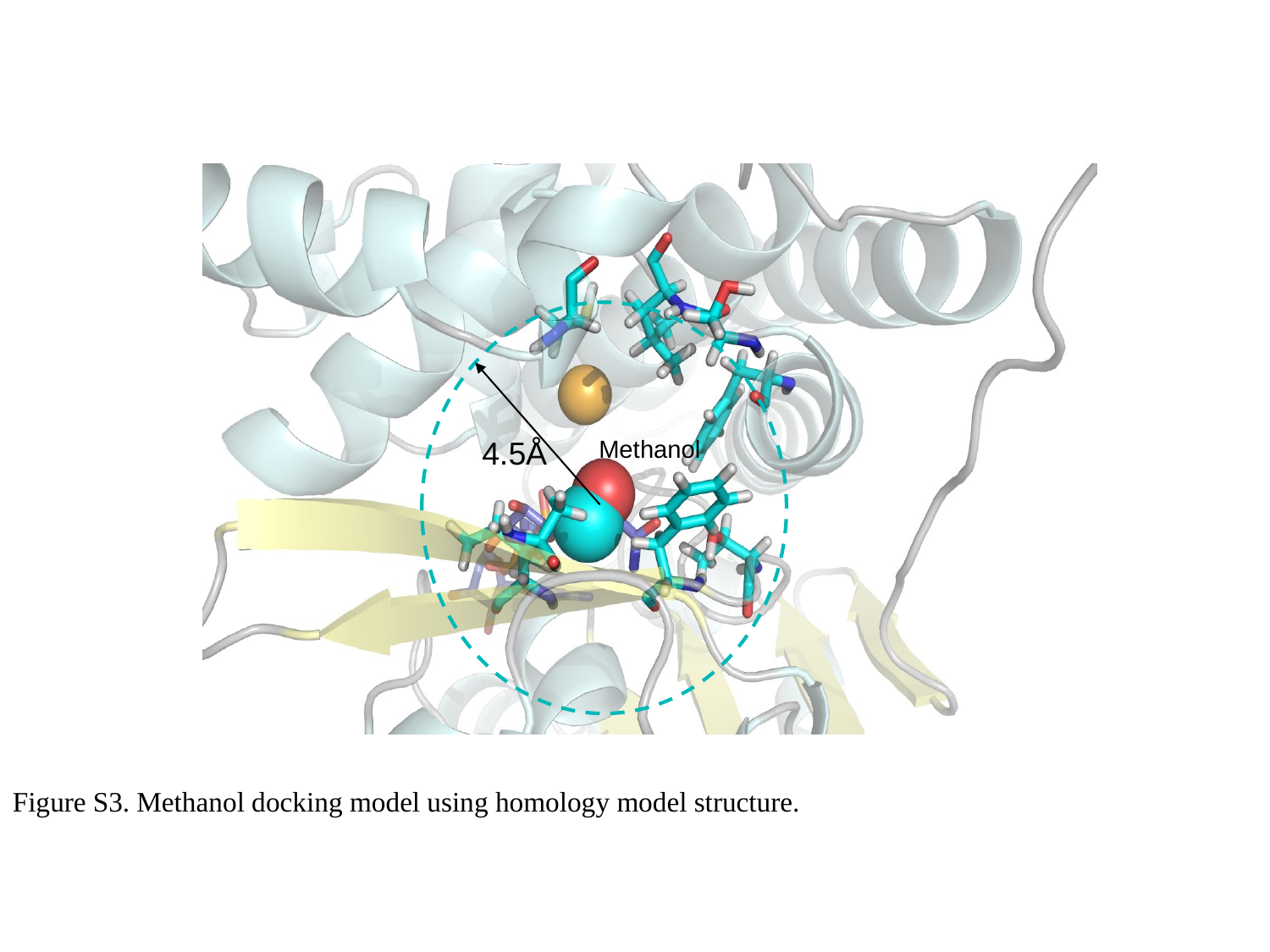

4.5Å
Methanol
Figure S3. Methanol docking model using homology model structure.

## Slide 4
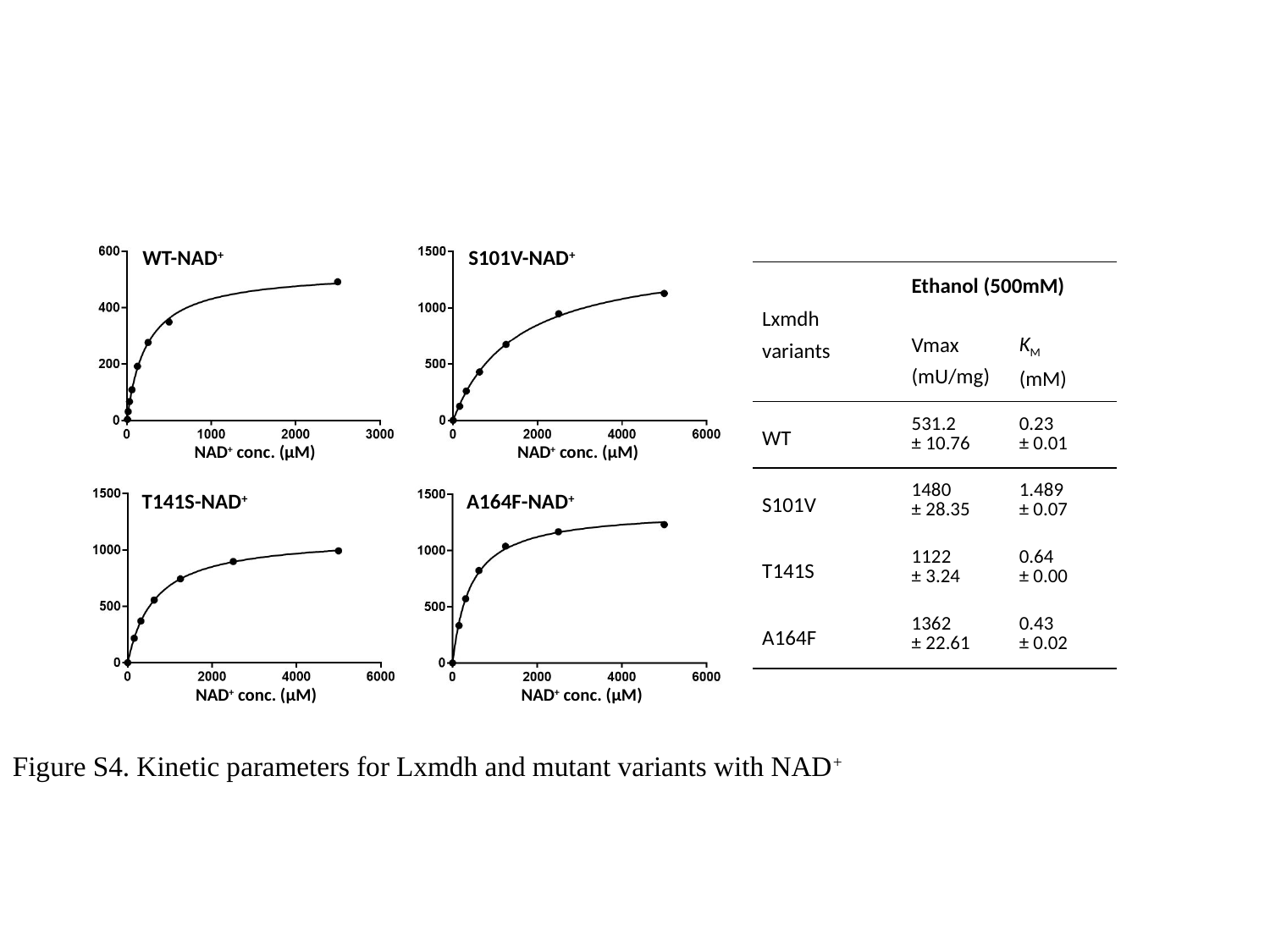

WT-NAD+
S101V-NAD+
| Lxmdh variants | Ethanol (500mM) | |
| --- | --- | --- |
| | Vmax (mU/mg) | KM (mM) |
| WT | 531.2 ± 10.76 | 0.23 ± 0.01 |
| S101V | 1480 ± 28.35 | 1.489 ± 0.07 |
| T141S | 1122 ± 3.24 | 0.64 ± 0.00 |
| A164F | 1362 ± 22.61 | 0.43 ± 0.02 |
NAD+ conc. (μM)
NAD+ conc. (μM)
T141S-NAD+
A164F-NAD+
NAD+ conc. (μM)
NAD+ conc. (μM)
Figure S4. Kinetic parameters for Lxmdh and mutant variants with NAD+
